# Neurological examination at 32-weeks postmenstrual age predicts 12-month cognitive outcomes in very preterm-born infants

**DOI:** 10.1038/s41390-022-02310-6

**Published:** 2022-09-23

**Authors:** Isabel U. Huf, Emmah Baque, Paul B. Colditz, Mark D. Chatfield, Robert S. Ware, Roslyn N. Boyd, Joanne M. George

**Affiliations:** 1 School of Health Sciences and Social Work, Griffith University, Brisbane, QLD Australia; 2 Menzies Health Institute Queensland, Griffith University, Brisbane, QLD Australia; 3 University of Queensland Centre for Clinical Research, The University of Queensland, Brisbane, QLD Australia; 4 Perinatal Research Centre, Royal Brisbane and Women’s Hospital, Brisbane, QLD Australia; 5 Queensland Cerebral Palsy and Rehabilitation Research Centre, Centre for Children’s Health Research, Faculty of Medicine, The University of Queensland, Brisbane, QLD Australia; 6 Physiotherapy Department, Queensland Children’s Hospital, Children’s Health Queensland Hospital and Health Service, Brisbane, QLD Australia

## Abstract

**Background:**

To determine the diagnostic accuracy of Hammersmith Neonatal Neurological Examination (HNNE) at 30–32 weeks postmenstrual age (PMA, ‘Early’) and term equivalent age (TEA) in infants born <31 weeks PMA to predict cognitive outcomes at 12 months corrected age (CA).

**Methods:**

Prospective cohort study of 119 infants (73 males; median 28.4 weeks gestational age at birth) who underwent Early and TEA HNNE. At 12 months CA, 104 participants completed Bayley Scales of Infant and Toddler Development, 3rd Edition, (Bayley-III). Optimum cut-off points for each HNNE subscale were determined to establish diagnostic accuracy for predicting adverse cognitive outcomes on the Bayley-III Cognitive Composite Scale (≤85).

**Results:**

The best diagnostic accuracy for HNNE total score at 30–32 weeks PMA predicting cognitive impairment occurred at cut-off ≤16.7 (sensitivity (Se) = 71%, specificity (Sp) = 51%). The Abnormal Signs subscale demonstrated the best balance of sensitivity/specificity combination (Se = 71%, Sp = 71%; cut-off ≤1.5). For HNNE at TEA, the total score at cut-off ≤24.5 had Se = 71% and Sp = 47% for predicting cognitive impairment. The Tone Patterns subscale demonstrated the strongest diagnostic accuracy at TEA (Se = 71%, Sp = 63%; cut-off ≤3).

**Conclusions:**

Early and TEA HNNE demonstrated moderate diagnostic accuracy for cognitive outcomes at 12-months CA in infants born <31 weeks gestational age.

**Clinical Trial Registration:** Australian New Zealand Clinical Trials Registry; Trial Registration Number: ACTRN12613000280707; web address of trial: http://www.ANZCTR.org.au/ACTRN12613000280707.aspx.

**Impact:**

- Early Hammersmith Neonatal Neurological Examination (HNNE) assessment at 30–32 weeks postmenstrual age has moderate diagnostic accuracy for cognitive outcomes at 12 months corrected age in infants born <31 weeks gestation.
- Early HNNE at 30–32 weeks has stronger predictive validity than HNNE at term equivalent age.
- Early HNNE may provide an early marker for risk-stratification to optimise the planning of post-discharge support and follow-up services for infants born preterm.

## Introduction

Up to 60% of very preterm-born infants (born < 32 weeks gestational age) experience clinically significant cognitive impairments.^1^ This can range from mild to severe intellectual disability, with a large number experiencing long-term cognitive problems into childhood and adolescence.^2,3^ Cognitive impairments include lower intelligence, information processing speed and executive function, and may be associated with lower educational outcomes in childhood, particularly in mathematics and reading ability.^3–5^ These cognitive deficits worsen with decreasing gestational age at birth and do not appear to improve as the infants grow and mature.^3,5–9^ Researchers have thus concluded that cognitive impairments observed in very preterm-born children are deficits rather than delays, and without intervention these children will not catch up to their term born peers.^3^

While early intervention programs administered in the first 12 months of life have demonstrated positive impacts on cognition in very preterm-born children during infancy and at preschool, these effects are not sustained long-term.^10,11^ Thus, there is a need to identify strategies for improving the long-term cognitive outcomes of very preterm-born infants. In recent years there has been investigation into the use of prognostic tools, early in the neonatal period, to identify very preterm-born infants who are at risk of poor cognitive outcomes, with the objective of using this information to provide targeted interventions to those infants who are at greatest risk. Neonatal Magnetic Resonance Imaging (MRI) for detecting brain injury in this population has gained support as a useful prognostic tool.^12–15^ Many regional hospitals and those in low- and middle-income settings do not have access to neonatal MRI, thus it is important to establish the diagnostic accuracy of more accessible tools such as a clinical neurological assessment.

The Hammersmith Neonatal Neurological Examination (HNNE) is widely used in both clinical and research settings to detect atypical neurological function in preterm and term-born infants up to three months post-term age.^16,17^ The HNNE has good interrater reliability and is validated for use in term-born infants and preterm-born infants at term equivalent age (TEA, 38–42 weeks postmenstrual age).^16,18,19^ Among moderate (born 32–33 weeks) and late (born 33–36 weeks) preterm-born populations, a lower HNNE total score at TEA is associated with increased probability for cognitive delay at two years, as determined by the Bayley Scales of Infant and Toddler Development, 3rd Edition (Bayley III) Cognitive Composite Scale.^20^ Recent investigation has established the diagnostic accuracy of the HNNE for an outcome of neurodevelopmental disability in a population of preterm-born infants born at ≤36 weeks gestation, assessed at a mean 36 weeks postmenstrual age (PMA) and at TEA.^21^ Diagnostic accuracy of the HNNE performed earlier than 36 weeks PMA in a population of preterm-born infants born <31 weeks PMA for later cognitive outcomes has not been explored.

It is important to explore the diagnostic accuracy of the HNNE in the youngest preterm-born infants because decreasing gestational age at birth is associated with poorer cognitive function later in life.^3^ Furthermore, most infants will be discharged home before they reach TEA. An Early HNNE can be administered while infants are still in hospital and, thus families do not have to return to hospital for testing at TEA. Furthermore, early prognostic testing opens a window for early interventions to be implemented and for undertaking early risk stratification and planning for further close monitoring. The aims of this study are to:

- Compare HNNE scores in preterm-born and term born populations.
- Investigate the sensitivity and specificity of the HNNE in very preterm-born infants born <31 weeks PMA when assessed at 30–32 weeks PMA (‘Early’ assessment) and at 40–42 weeks PMA (‘TEA’ assessment) to predict cognitive outcomes on the Bayley III Cognitive Composite Scale at 12 months corrected age (CA).

## Methods

### Study design and participants

This diagnostic accuracy study forms part of a larger prospective cohort study investigating earlier biomarkers to predict neurodevelopmental outcomes of children born very preterm, the PPREMO (Prediction of PREterm Motor Outcomes) study.^22^ The preterm-born cohort were recruited from the Neonatal Intensive Care Unit at the Royal Brisbane and Women’s Hospital, Brisbane, Australia. Before being formally enroled in the study, parents or guardians of the infants provided informed written consent. Infants born at <31 weeks PMA, whose family lived within 200 km of the hospital and spoke English were eligible for the study. Infants with congenital or chromosomal abnormalities were excluded. Socio-demographic information was collected from participants’ families to identify higher social risk. Social risk was assessed using a score measuring six aspects of social status including: family structure, education of primary caregiver, occupation of primary income earner, employment status of primary income earner, language spoken at home, and maternal age.^23,24^ Each item was scored between 0 and 2 for a total score of 12. Scores ≥2 are considered high social risk as per existing studies in this population.^24^ Ethical approval for this study was granted by the Human Research and Ethics Committee at The Royal Brisbane and Women’s Hospital (HREC/12/QRBW/245), and The University of Queensland (UQ, 2012001060). The study has been registered with the Australian New Zealand Clinical Trials Registry (ACTRN12613000280707).

The reference sample of 46 healthy term-born infants were recruited from the postnatal ward or as volunteered by their caregiver by word of mouth. Eligibility criteria for the healthy term reference group included participation in one of three studies: PPREMO;^22^ the PREterm Brain Outcomes study (PREBO: Children’s Health Queensland [HREC/15/QRCH/7], UQ [2015000290] and ACTRN12615000591550);^25^ or the PREterm infant Massage by the Mother study (PREMM: RBWH [HREC/09/QRBW/296], Children’s Health Queensland [HREC/12/QRCH/40], UQ [2014001160] and ACTRN12612000335897).^26^ Eligibility criteria included: birth between 38- and 41-weeks PMA; absence of pregnancy and/or birth complications; birth weight above the 10th percentile; and no admission to a neonatal intensive care or special care unit after birth.^22^

### Hammersmith Neonatal Neurological Examination (HNNE)

The HNNE consists of 34 items, grouped in six subscales: Posture and Tone, Tone Patterns, Reflexes, Movements, Abnormal Signs, and Orientation and Behaviour. Each item receives a raw score between 1 and 5.^17^ Raw scores are converted to optimality scores based on the distribution of scores in typically developing term-born infants.^16,19,27,28^ Scores above the 10th percentile receive a score of 1; scores falling between the 10th and 5th percentile receive a score of 0.5; and scores below the 5th percentile are scored 0.^16,19,27^ These scores are summed to obtain a global optimality score for a maximum possible total of 34.^16^

Preterm-born infants underwent Early and TEA HNNE conducted by a single clinical assessor, masked to infant medical history.^22^ At the Early timepoint, the Placing item of the HNNE Reflexes subscale was not administered as infants were assessed in incubators without sufficient room to administer the item. The Reflexes subscale and total HNNE scores were adjusted by adding 0.905 to each infants’ scores to reflect average scores achieved by infants on the Placing item of the Reflexes subscale from existing, published preterm data.^29^ Additionally, in some cases the Early HNNE could not be completed due to respiratory equipment rendering some items unable to be administered. In these cases, some subscale scores were still available, but a total score could not be calculated. The term-born reference sample were examined on the HNNE at 40–42 weeks PMA.

### The Bayley Scales of Infant and Toddler Development, 3rd Edition (Bayley III)

At 12 months CA, children were assessed by a single clinician trained in the Bayley III and masked to HNNE scores and medical history. The Bayley III is a widely used assessment that measures the developmental functioning of infants and toddlers aged one month to 42 months.^30^ The Cognitive Composite score was utilised in this study with a published mean (SD) score of 100 (15).^31^ The assessment is norm referenced to a sample of American children including 10% with developmental impairment.^32^ Consequently, in populations of preterm-born children, the Bayley III’s normative criteria underestimates the proportion of children with cognitive impairment.^32–34^ To accommodate for this underestimation of cognitive impairment in the normative data, scores ≤ –1SD were considered atypical.^31,32,35^

### Statistical analysis

Demographic and clinical assessment data were analysed and presented in the form of mean (SD) and median (IQR) along with range (min–max) for continuous data and frequency (percentage) for categorical data. Early and TEA HNNE data were presented graphically using a box and whisker plot. The Early and TEA HNNE data were dichotomised using the 5th percentile value of the term-born reference sample as the cut-off point, for each of the subscales and total scores. The Bayley III scores were dichotomised using –1SD (i.e., Bayley III score ≤85) as the cut-off point. Two by two tables were constructed to derive diagnostic accuracy statistics. Receiver operating characteristic (ROC) curves were used to determine HNNE cut-off points that maximised sensitivity (Se) and specificity (Sp) whereby the HNNE subscale and total scores were the test variables and the dichotomised Bayley III score was the state variable. Analyses were performed using Stata statistical software, Version 16 (StataCorp, College Station, TX).

## Results

### Participants and clinical assessment data

One hundred and nineteen preterm infants underwent Early HNNE assessment and 107 underwent TEA assessment. Of these infants, 109 had complete Early HNNE data, and 104 had complete TEA data. One hundred and four infants returned for 12 month follow up. Participant characteristics for all preterm-born infants are summarised in Table 1. The median (IQR) PMA at birth for the preterm-born sample (*n* = 119) and term-born reference sample (*n* = 46) was 28.4 (26.9–29.4) weeks and 39.9 (39.0–40.4) weeks, respectively. Demographic details for the 104 infants with outcome data available and the 15 without are presented in Supplementary Table 1. Preterm-born infants without outcome data had significantly larger head circumference, birth weight, and fewer continuous positive airway pressure days. Table 2 presents clinical assessment data for all preterm-born infants as well as the term-born reference sample. Early HNNE assessment occurred at a median (IQR) 31.9 (31.0–33.3) weeks PMA. Median (IQR) age at preterm-born TEA assessment was 40.4 (40.0–41.3) weeks PMA. Mean (SD) age at Bayley III assessment was 52.7 (51.7–53.6) weeks CA. HNNE scores for infants with and without outcome data are presented in Supplementary Table 2. Children who did not return for follow up had significantly higher scores on the Tone Patterns subscale (*p* = 0.02). There were no other significant differences in HNNE scores between these groups.

**Table 1:** Characteristics of the study sample.

Birth and maternal data	Preterm group	Term-born control
	*n* = 119	*n* = 46
PMA at birth (weeks)	28.4 (26.9–29.4)	39.9 (39.0–40.4)
Birth weight (g)	1093 (321)	3513 (314)
Birth head circumference (cm)	25.8 (2.4)	34.7 (1.1)
Males	73 (61%)	23 (52%)
Multiple births	36 (30%)	0 (0%)
Premature rupture of membranes	27 (23%)	4 (12%)
Caesarean section	84 (71%)	
Chorioamnionitis	18 (15%)	
Antenatal steroids	83 (70%)	
Magnesium sulphate	63 (64%)	
Higher social risk^a^	58 (49%)	5 (15%)
*Acquired medical factors*		
Patent ductus arteriosus	59 (50%)	
Any intraventricular haemorrhage	30 (25%)	
Intraventricular haemorrhage grade III or IV	8 (7%)	
Periventricular leukomalacia	4 (3%)	
Hydrocephalus^b^	4 (3%)	
Seizures treated with anticonvulsant therapy	1 (1%)	
NEC diagnosed or suspected	5 (4%)	
Confirmed sepsis	5 (4%)	
Total parenteral nutrition (days)	11.5 (8.5–15.0)	
Postnatal corticosteroids	20 (17%)	
Ventilation (days)	2.0 (0.0–11.0)	
CPAP (days)	25.5 (7.0–44.0)	
Oxygen therapy (h)	49 (2–444)	
Bronchopulmonary dysplasia^c^	34 (29%)	

Data are presented as mean (SD) or median (IQR); range min–max for continuous measures and *n* (%) for categorical measures.

*CPAP* continuous positive airway pressure, ‘Early’ refers to clinical assessment between 30 and 32 weeks postmenstrual age. *NEC* necrotising enterocolitis. *PMA* postmenstrual age. ‘TEA’ refers to clinical assessment at term equivalent age, 40–42 weeks postmenstrual age.

^a^Higher social risk is defined as social risk score of 2 or above.

^b^All 3 infants with hydrocephalus also had IVH grade III/IV.

^c^defined as oxygen requirement at 36 weeks.

**Table 2:** Clinical assessment data for preterm-born infants and term-born reference sample.

		Preterm-born infants	Term-born reference sample
		*n* = 119	*n* = 46
PMA at Early HNNE (weeks)		31.9 (31.0–33.3)	
PMA at TEA HNNE (weeks)		40.4 (40.0–41.3)	41.3 (40.9–42.3)
*HNNE optimality subscale and total scores*			
Posture and Tone	Early	3.8 (1.9), *n* = 111	8.7 (1.4)
	TEA	7.0 (1.6)	
Tone Patterns	Early	3.9 (0.8), *n* = 111	4.3 (0.8)
	TEA	3.6 (0.8)	
Reflexes	Early	2.4 (1.0), *n* = 113	5.3 (0.6)
	TEA	4.1 (1.1)	
Spontaneous Movements	Early	1.0 (0.8), *n* = 110	2.8 (0.4)
	TEA	2.3 (0.8)	
Abnormal Signs	Early	2.0 (0.6)	2.8 (0.2)
	TEA	2.6 (0.5)	
Orientation and Behaviour	Early	2.9 (1.5)	6.1 (0.9)
	TEA	5.2 (1.2)	
Total score	Early	16.2 (3.7), *n* = 109	30.1 (2.6)
	TEA	24.8 (3.7)	
*Bayley III cognitive composite score*			
CA at assessment (weeks)		52.7 (51.7–53.6)	
Bayley III Cognitive Composite score		105.0 (97.5–110.0)	

Data are presented as mean (SD) or median (IQR).

*CA* corrected age. ‘Early’ refers to clinical assessment at 30–32 weeks postmenstrual age. *HNNE* Hammersmith Neonatal Neurological Examination, *PMA* postmenstrual age, ‘TEA’ refers to term equivalent age.

### HNNE scores for all preterm-born infants compared to term-born infants’ scores

**Fig. 1:**
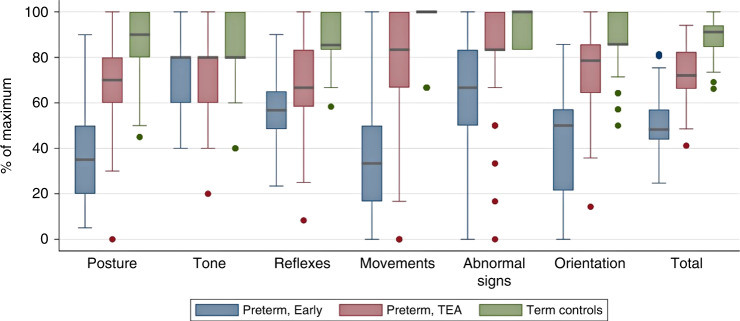
Distribution of HNNE scores for preterm-born and term-born reference samples. Blue indicates Early HNNE, pink indicates TEA HNNE, green indicates term reference sample; thick line inside box indicates median score; whiskers indicate minimum and maximum scores; ‘Early’ refers to clinical assessment at 30–32 weeks postmenstrual age; HNNE Hammersmith Neonatal Neurological Examination. ‘TEA’ refers to term equivalent age.

The distribution of HNNE subscale and total scores for preterm-born infants and term born reference sample are presented in Fig. 1. Except for the Tone Patterns subscale, preterm born infants achieved lower median scores at both the Early and TEA assessments across all subscales and total score. Within the preterm-born group, median Early assessment scores were lower than TEA assessment scores. The preterm-born group also displayed a wider range of scores across all subscales and total score compared to the term-born reference sample.

### Diagnostic accuracy of HNNE predicting cognitive outcome

The sensitivity, specificity, and accuracy of Early and TEA HNNE assessment to predict Bayley III Cognitive Composite scores ≤85 at 12-months CA, using the 5th percentile values of the term control data as cut-off points are presented in Table 3. At Early HNNE assessment, the Reflexes subscale had the strongest predictive value (Se 100%, Sp 21%, Positive Predictive Value PPV 9%, Negative Predictive Value NPV 100%, ≤4 cut-off). The TEA Tone Patterns subscale demonstrated the best combination of sensitivity and specificity (Se 71%, Sp 63%, PPV 13% NPV 93%, cut-off ≤3).

**Table 3:** Sensitivity, specificity and accuracy of Early and TEA HNNE assessment for predicting Bayley III Cognitive Composite score ≤ 85 at 12 months using cut-off points derived from term reference sample 5th percentile values.

Subscale	Age at assessment	Cut point	Se (%)	95% CI	Sp (%)	95% CI	CC (%)	95% CI	AUC	95% CI	PPV (%)	95% CI	NPV (%)	95% CI
Posture and Tone	Early	5.5	86	42.1–99.6	16	8.9–25.0	21	8.0–30	0.5	0.4–0.6	7	2.8–15.4	93	68.1–99.8
	TEA		14	0.4–57.9	83	73.3–89.7	78	60.7–95.3	0.6	0.3–0.6	6	0.15–28.7	93	84.8–97.3
Tone Patterns	Early	3	43	9.9–81.6	66	55.5–76.0	65	48.9–81.0	0.5	0.3–0.7	9	1.9–24.3	94	84.5–98.2
	TEA		71	29.0–96.3	63	52.3–72.9	64	48.3–79.7	0.7	0.5–0.9	13	4.3–27.4	97	88.5–99.6
Reflexes	Early	4	100	59.0–100.0	21	13.1–30.7	27	16.8–37.2	0.6	0.6–0.7	9	3.6–17.4	100	82.4–100.0
	TEA		43	9.9–81.6	41	31.1–52.1	41	28.6–53.4	0.4	0.2–0.6	5	1.1–14.6	90	77.4–97.3
Spontaneous Movement	Early	2	100	59.0–100.0	9	4.0–17.1	16	8.2–23.8	0.5	0.5–0.6	8	3.3–15.9	100	63.1–100.0
	TEA		57	18.4–90.1	55	44.7–65.8	56	41.3–70.7	0.6	0.3–0.8	9	2.4–21.2	94	84.6–98.8
Abnormal Signs	Early	2.5	100	59.0–100.0	4	1.0–10.2	11	4.8–17.2	0.5	0.5–0.5	7	2.9–13.9	100	39.8–100.0
	TEA		71	29.0–96.3	39	29.1–49.9	41	28.6–53.4	0.6	0.4–0.7	8	3.7–18.1	95	82.3–99.4
Orientation and Behaviour	Early	4	100	59.0–100.0	13	7.3–21.8	19	11.9–28.9	0.6	0.5–0.6	8	3.1–15.2	100	75.3–100.0
	TEA		14	0.4–57.9	76	66.1–84.4	72	55.4–88.6	0.4	0.3–0.6	4	0.1–21.9	92	83.6–97.0
Total score	Early	25	100	59.0–100.0	2	0.3–8.0	10	2.8–15.2	0.5	0.5–0.5	8	3.1–15.1	100	15.8–100.0
	TEA		71	29.0–96.3	40	30.1–51.0	42	29.5–54.5	0.5	0.4–0.7	8	2.8–18.4	95	82.7–99.4

*AUC* area under the curve, *CC* correctly classified. ‘Early’ refers to clinical assessment between 30 and 32 weeks postmenstrual age. *HNNE* Hammersmith Neonatal Neurological Examination, *NPV* negative predictive value, *PPV* positive predictive value, *Se* sensitivity, *Sp* specificity. ‘TEA’ refers to clinical assessment at term equivalent age. *95% CI* 95% confidence interval.

### Area under the curve analyses to determine HNNE cut-off points with the best predictive ability for cognitive outcomes

The Early HNNE assessment had slightly better predictive ability for cognitive outcomes than the TEA HNNE. Analysis of the ROC curves (Table 4 and Supplementary Table 3) revealed that the Abnormal Signs subscale at the Early time point produced the strongest combination of sensitivity and specificity for predicting cognitive impairment at 12 months on the Bayley III (Se 71%, Sp 71%, ≤1.5 cut-off). Total optimality scores at Early assessment led to Se 71%, Sp 51%, ≤17 cut-off. At TEA, the HNNE Tone Patterns subscale demonstrated the best combination of sensitivity and specificity (Se 71%, Sp 63%, ≤3 cut-off) for predicting cognitive impairment on the Bayley III Cognitive Composite Scale. The TEA total HNNE optimality score had Se 71%, Sp 47%, ≤24 cut-off.

**Table 4:** Sensitivity, specificity, and accuracy of Early and TEA HNNE assessment for predicting Bayley III Cognitive Composite score ≤ 85 at 12 months using cut-off points derived from ROC curve analysis.

Subscale	Age at assessment	Cut point	Sensitivity (%)	95% Confidence interval	Specificity (%)	95% Confidence interval	% Correctly classified	95% Confidence interval
Posture and Tone	Early	≤3.75	71	29.0–96.3	51	39.8–61.3	52	37.7–66.3
	TEA	≤7	71	29.0–96.3	41	31.1–52.1	43	28.8–53.2
Tone Patterns	Early	≤4	71	29.0–96.3	21	13.4–31.3	25	15.2–33.8
	TEA	≤3	71	29.0–96.3	63	52.3–72.9	64	48.3–79.7
Reflexes	Early	≤3	86	42.1–47.4	58	47.4–68.5	60	44.8–75.2
	TEA	≤5	86	42.1–99.6	22	13.8–31.6	26	16.2–35.8
Spontaneous Movement	Early	≤1	86	42.1–99.6	37	27.4–48.5	41	29.3–54.7
	TEA	≤2.5	86	42.1–99.6	41	31.1–52.1	44	31.2–56.9
Abnormal Signs	Early	≤1.5	71	29.0–96.3	71	61.0–79.9	71	54.7–87.3
	TEA	≤2.5	71	29.0–96.3	39	29.1–49.9	41	29.7–53.5
Orientation and Behaviour	Early	≤3.5	71	29.0–96.3	37	27.5–47.5	39	30.0–68
	TEA	≤5.5	71	29.0–96.3	42	32.1–53.1	44	31.1–56.8
Total score	Early	≤17	71	29.0–96.3	51	39.6–61.5	52	37.6–66.4
	TEA	≤24	71	29–96.3	47	36.3–57.4	48	34.6–61.4

*CC* correctly classified. ‘Early’ refers to clinical assessment between 29 and 35 weeks postmenstrual age. *HNNE* Hammersmith Neonatal Neurological Examination, *ROC* receiver operated characteristic, *Se* sensitivity, *Sp* specificity. ‘TEA’ refers to term equivalent age. *95% CI* 95% confidence interval.

Higher scores indicate better neurological function.

## Discussion

The HNNE performed Early and at TEA in infants born <31 weeks PMA, demonstrated moderate diagnostic accuracy for cognitive outcomes at 12 months CA. This study provides the first HNNE cut-off points specific to a preterm-born population born <31 weeks PMA to predict cognitive impairment at 12 months CA and found that Early HNNE, particularly the Abnormal Signs subscale, is at least as predictive as TEA HNNE in this cohort of infants. This finding is important as many preterm-born infants will be discharged to home or transferred from tertiary hospitals before they reach TEA and may consequently be lost to follow up. The clinical implications are that early neurological testing can be valuable for screening and planning for further monitoring and for baseline assessment prior to early intervention. Furthermore, early prognostic screening can identify those very preterm-born infants who will benefit from early interventions to improve their cognitive outcomes.

Compared to the term-born reference sample, the preterm-born group achieved lower mean subscale and total scores on both the Early and TEA assessments. This is a pattern that has been previously identified by researchers. A study from Brown et al. in 2006 examined 168 infants born <30 weeks with the HNNE at term age, who also achieved lower mean subscale and total scores compared to the term-born group.^36^ The disparity between preterm-born and term-born HNNE scores tends to lessen with increasing gestational age.^20^

The subscale which had the best combination of sensitivity and specificity was Abnormal Signs at the Early assessment. The strength of this finding may be explained by previously published normative data for TEA HNNE among preterm-born infants.^19^ Findings from this previously published study showed that differences in the range of scores and median scores between preterm-born and term-born infants were most common in the Abnormal Signs subscale.^19^ This was especially true for the tremors and startles items of Abnormal Signs subscale in infants born at 25–27 weeks.^19^ Twenty-five percent of the present preterm-born group were born ≤27 weeks PMA hence this may account for the strong sensitivity and specificity of the Abnormal Signs subscale for cognitive impairment at 12 months CA. Additionally, when other subscales were unable to be administered, the Abnormal Signs subscale was usually able to be completed and it thus had the highest rate of completed data.

The finding in the present study that Early and TEA HNNE has predictive validity for cognitive outcomes at 12 months CA are supported by those from Spittle et al. in 2017. This study revealed that in a population of moderate-preterm-born (born 32–33 weeks PMA) and late-preterm-born infants (born 34–36 weeks PMA), HNNE total score ≤10th percentile at TEA was associated with increased odds of cognitive delay at two years, as determined by Bayley III.^20^ Their preterm-born group had a mean (SD) gestational age at birth of 34.4 (1.2) weeks and mean (SD) total optimality score of 29.7 (2.6). The moderate-preterm-born infants had a mean (SD) gestational age at birth of 33 (0.6) weeks PMA and scored a mean (SD) HNNE total score of 30.4 (2.43). Late-preterm-born infants were born at a mean (SD) gestational age of 35.2 (0.8) weeks PMA and scored 30.42 (2.62). In contrast, the present sample of very-preterm-born infants achieved lower mean (SD) HNNE total scores at both the Early (16.2 [3.7]) and TEA (24.8 [3.7]) timepoints, which is explained by the earlier median (IQR) gestational age at birth (28.4 [26.9–29.4]), and younger mean (SD) PMA at assessment for the Early assessment (32.4 [1.5]). This result is expected as decreasing gestational age at birth is associated with poorer scores on the HNNE.^3^

Prior studies relating to the present sample of preterm-born infants found associations between Early TEA HNNE scores and two-year neurodevelopmental outcomes.^12^ The present study now extends that work by providing diagnostic accuracy statistics for subscales and overall HNNE scores. We previously demonstrated that the Reflexes subscale was strongly associated with cognitive outcomes at two-years.^12^ This supports the finding in the present study that the HNNE Reflexes subscale has strong sensitivity for predicting cognitive impairment at 12 months CA (Se 85.7%, Sp 58.2%; cut-off ≤3). These findings highlight the usefulness of an Early HNNE as a predictive tool in very preterm born infants.

This study supports previous findings for the use of the HNNE early in the neonatal period to predict future outcomes for infants born very preterm. A study by Venkata et al. used ROC curve analysis to identify the first HNNE optimality cut-off points prior to term-age and at term-age HNNE to predict neurodevelopmental disability at 12 months CA. This was defined as a score of <70 on the Indian adapted Bayley III, motor delay with neurological signs, presence of seizures, requirement of hearing aid, blindness in one or both eyes.^21^ Their preterm-born group of infants born ≤36 weeks PMA included 30% born ≤32 weeks PMA (mean age at preterm-age assessment was 36.1 weeks PMA).^21^ All preterm-born infant age groups were combined to determine the most predictive HNNE composite optimality cut-off point prior to term-age HNNE. It was found that a cut-off point of ≤32.5 produced similar results both before and at TEA (Se 64%, Sp 73%; and Se 50%, Sp 77%, respectively).^21^ The present study builds on these important findings, assessing infants four weeks earlier, performing analyses for each subscale individually and using a specific outcome of cognition rather than a composite neurodevelopmental outcome. Our study demonstrated that Early HNNE is at least as effective as TEA HNNE, to predict an outcome of cognitive impairment at 12 months CA. In addition, the Early HNNE Abnormal Signs subscale (cut-off ≤1.5) provided the best balance of sensitivity (71.4%) and specificity (71.1%) to predict cognitive impairment at 12 months CA in infants born <31 weeks.

The results of the present study are strengthened by limiting the outcome measure to cognitive impairment as identified by the Bayley III Cognitive Composite Scale, whereas other studies have used several outcome measures. Additionally, the present study performed analyses for all HNNE subscales both Early and TEA, enabling the identification of subscales that have particularly useful diagnostic accuracy statistics. A final strength of the present study is that it establishes the first Early HNNE cut-off points for cognitive outcomes within a very preterm-born population born <31 weeks PMA.

Our findings suggest that some subscales of the Early and TEA HNNE have better diagnostic accuracy for an outcome of cognitive impairment compared to General Movements Assessment (GMA) administered during the writhing period (birth to 6–9 weeks CA). General Movements Assessment is an existing clinical early prediction tool that has been closely investigated and at term-age among infants born <30 weeks PMA, has 64% sensitivity and 57% specificity for a suspect or atypical cognitive outcome on the Bayley Scales of Infant and Toddler Development, 2nd Edition.^37,38^ At one month post-term age, GMA has 80% sensitivity and 41% specificity for moderate to severe cognitive impairment on the Bayley III.^39^ Thus, Early and TEA HNNE has better diagnostic accuracy and can be administered earlier than GMA. The HNNE is also favourable as it does not require expensive training and resources, unlike GMA. Other clinical tools including the NICU Neonatal Neurobehavioral Scale (NNNS) have poor clinical utility due to complex administration, scoring and interpretation.^18^ Objective tools to measure brain structure like neonatal MRI are costly and only available to a small number of infants with limited access. In contrast, the HNNE is a simple, inexpensive, standardised measure of neurological function with good clinical utility, making it an ideal predictive tool for smaller hospitals and in low resource settings.

The follow up age of 12 months CA limits interpretation of the present results. Follow up at 12 months CA is too early to definitively determine cognitive outcomes. Follow-up into childhood would enable more concrete conclusions around cognitive outcomes and the predictive validity of early neurological assessment. Data collection is ongoing in the broader study of outcomes at two years (PREBO: NHMRC 1084032) and six years (PREBO-6: NHMRC 1161998). Cut-offs presented in this study will need to be validated in independent samples.

To determine the best early screening tools for use in a very preterm-born population, the diagnostic accuracy of other clinical neurological assessments must be investigated in this population, and at the same time-points. Assessments that can be used in this population include the NNNS, Premie-Neuro and the Test of Infant Motor Performance.^36,40^ Additionally, randomised controlled trials where infants undergo early prognostic screening via clinical neurological assessment prior to interventions will enable researchers to target infants who may benefit. This will lead to improved understanding around the efficacy of early interventions to improve cognitive outcomes.

## Conclusion

Very early neonatal assessment opens a new window for early interventions to be implemented with the aim of improving cognitive outcomes for very preterm-born infants. Early HNNE may provide an early marker for risk-stratification to optimise the planning of post-discharge support and follow-up services. As most preterm-born infants will be discharged from hospital before TEA, early assessment also means that families do not need to return to the hospital for assessment and are less likely to be lost to follow-up.

Ultimately, the results of this prospective cohort study suggest that the combination of Early HNNE scores with other clinical and neuroimaging data will likely provide more accurate identification of those infants at greatest risk of adverse cognitive outcomes.

## Supplementary Information

Supplementary table

## Data Availability

The datasets generated during and/or analysed during the current study are available from the corresponding author on reasonable request.
