# Supplementary material for: Neurological examination at 32-weeks postmenstrual age predicts 12-month cognitive outcomes in very preterm-born infants
Source: Pediatr Res. 2022 Sep 23;93(6):1721–7. doi: 10.1038/s41390-022-02310-6 (PMC10172122; doi:10.1038/s41390-022-02310-6)
Supplement: Supplementary file 1 — Supplementary table [file 41390_2022_2310_MOESM1_ESM.pdf]

Supplementary Table 1: Characteristics of the preterm-born infants with cognitive outcome and without cognitive outcome. Data are presented as mean (SD) or median (IQR); range min-max for continuous measures and n (%) for categorical measures.

**Birth and Maternal Data**	Preterm-born infants with cognitive outcome **n=104**	Preterm-born infants without cognitive outcome **n=15**	**p-value**
PMA at birth (weeks)	28.3 (26.7-29.4)	28.9 (28.1-29.6)	0.20
Birth weight (g)	1059 (308)	1327 (327)	**<0.01**
Birth head circumference (cm)	25.6 (2.3)	27.0 (2.5)	**0.04**
Males	61 (59%)	12 (80%)	0.16
Multiple births	34 (33%)	2 (13%)	0.23
Premature rupture of membranes	23 (22%)	4 (27%)	0.74
Caesarean section	75 (72%)	9 (60%)	0.37
Chorioamnionitis	17 (16%)	1 (7%)	0.46
Antenatal steroids	76 (73%)	7 (47%)	0.07
Magnesium sulphate	56 (65%)	7 (58%)	0.75
Higher social risk$	50 (48%)	8 (57%)	0.58
**Acquired medical factors**			
Patent ductus arteriosus	52 (50%)	7 (47%)	1.00
Any intraventricular haemorrhage	24 (23%)	6 (40%)	0.20
Intraventricular haemorrhage grade III or IV	7 (7%)	1 (7%)	1.00
Periventricular leukomalacia	4 (4%)	0 (0%)	1.00
Hydrocephalus*	3 (3%)	1 (7%)	0.42
Seizures treated with anticonvulsant therapy	1 (1%)	0 (0%)	1.00
NEC diagnosed or suspected	4 (4%)	1 (7%)	0.50
Confirmed sepsis	4 (4%)	1 (7%)	0.50
Total parenteral nutrition (days)	11.0 (9.0-15.0)	12.5 (6.5-14.0)	0.70
Postnatal corticosteroids	19 (18%)	1 (7%)	0.46
Ventilation (days)	3.0 (0.5-15.5)	2.0 (0.0-6.0)	0.30
CPAP (days)	30.5 (7.5-46.5)	11.0 (7.0-17.0)	**0.02**
Oxygen therapy (hours)	56 (2-512)	37 (5-405)	0.83
Bronchopulmonary dysplasia#	31 (30%)	3 (20%)	0.55

Key: *CPAP* continuous positive airway pressure, 'Early' refers to clinical assessment between 30 – 32 weeks postmenstrual age, *NEC* necrotizing enterocolitis, *PMA* postmenstrual age, 'TEA' refers to clinical assessment at term equivalent age; $ Higher social risk is defined as social risk score of 2 or above; #defined as oxygen requirement at 36 weeks; *All 3 infants with hydrocephalus also had IVH grade III/IV.

Supplementary Table 2: Clinical assessment data for preterm-born infants with cognitive outcome and without cognitive outcome. Data are presented as mean (SD) or median (IQR).

		Preterm-born infants with cognitive outcome **n=104**	Preterm-born infants without cognitive outcome **n=15**	**p-value**
PMA at Early HNNE (weeks)		32.4 (1.5)	31.7 (1.1)	0.07
PMA at TEA HNNE (weeks)		40.6 (40.0-41.4)	40.0 (40.0-40.3)	0.08
**HNNE Optimality Subscale and Total Scores**				
Posture and tone	Early	3.8 (1.8)	3.7 (2.4)	0.89
	TEA	6.9 (1.6)	7.7 (1.7)	0.17
Tone patterns	Early	3.8 (0.8)	4.3 (0.5)	**0.02**
	TEA	3.6 (0.9)	3.8 (0.7)	0.63
Reflexes	Early	2.4 (1.0)	2.5 (1.1)	0.92
	TEA	4.1 (1.1)	4.3 (1.2)	0.77
Spontaneous movements	Early	1.0 (0.8)	1.1 (0.9)	0.88
	TEA	2.2 (0.8)	2.6 (0.6)	0.26
Abnormal signs	Early	2.0 (0.6)	2.1 (0.6)	0.46
	TEA	2.6 (0.5)	2.6 (0.5)	1.00
Orientation and behavior	Early	2.9 (1.5)	3.0 (1.6)	0.82
	TEA	5.3 (1.2)	4.7 (1.7)	0.17
Total	Early	16.1 (3.5)	16.8 (4.8)	0.52
	TEA	24.7 (3.7)	25.5 (4.2)	0.55
**Bayley III Cognitive Composite Score**				
CA at assessment (weeks)		52.7 (51.7-53.6)		
Bayley III Cognitive Composite score		105.0 (97.5-110.0)		

Key: *CA* corrected age, 'Early' refers to clinical assessment between 30-32 weeks postmenstrual age, *HNNE* Hammersmith Neonatal Neurological Examination, *PMA* postmenstrual age, 'TEA' refers to clinical assessment at term equivalent age.

Supplementary Table 3: Sensitivity, specificity, and accuracy of Early and TEA HNNE assessment for predicting Bayley III Cognitive Composite score ≤85 at 12 months using cut-off points derived from ROC curve analysis.

Subscale	Age at Ax	Cut-off point	Se (%)	95% CI	Sp (%)	95% CI	CC (%)	95% CI	AUC	95% CI	PPV (%)	95% CI	NPV (%)	95% CI
Posture and tone	Early	≤3.7	71	29.0-96.3	51	39.8-61.3	52	37.7-66.3	0.6	0.42-0.8	10	3.4-22.2	96	85.5-99.5
	TEA	≤7	71	29.0-96.3	41	31.1-52.1	43	28.8-53.2	0.6	0.4-0.7	8	2.8-18.7	95	83.1-99.0
Tone patterns	Early	≤4	71	29.0-96.3	21	13.4-31.3	25	15.2-33.8	0.5	0.3-0.6	7	2.2-14.9	90	69.6-98.8
	TEA	≤3	71	29.0-96.3	63	52.3-72.9	64	48.3-79.7	0.7	0.5-0.9	13	4.3-27.4	97	88.5-99.6
Reflexes	Early	≤3	86	42.1-47.4	58	47.4-68.5	60	44.8-75.2	0.7	0.6-0.9	14	1.2-27.4	98	90.1-100.0
	TEA	≤5	86	42.1-99.6	22	13.8-31.6	26.3	16.2-35.8	0.5	0.4-0.7	8	2.9-16	95	76.2-99.9
Spontaneous movement	Early	≤1	86	42.1-99.6	37	27.4-48.5	41	29.3-54.7	0.6	0.5-0.8	10	3.7-20.3	97	84.7-99.9
	TEA	≤2.5	86	42.1-99.6	41	31.1-52.1	44.4	31.2-56.9	0.6	0.5-0.8	10	3.8-20.5	97	86.5-99.9
Abnormal signs	Early	≤1.5	71	29.0-96.3	71	61.0-79.9	71	54.7-87.3	0.7	0.5-0.9	15	5.1-31.9	97	90.2-99.7
	TEA	≤2.5	71	29.0-96.3	39	29.1-49.9	41	29.7-53.5	0.6	0.4-0.7	8	2.7-18.1	95	82.3-99.4
Orientation and behaviour	Early	≤3.5	71	29.0-96.3	37	27.5-47.5	39	30.0-68	0.5	0.37-0.7	8	2.5-16.8	95	82.3-99.4
	TEA	≤5.5	71	29.0-96.3	42	32.1-53.1	44	31.1-56.8	0.6	0.4-0.7	9	2.9-19.0	95	83.5-19.0
Total score	Early	≤16.6	71	29.0-96.3	51	39.6-61.5	52	37.6-66.4	0.6	0.4-0.8	10	3.5-22.7	96	85.2-99.5
	TEA	≤24.5	71	29 – 96.3	47	36.3-57.4	48	34.6-61.4	0.6	0.4-0.8	9	3.1-20.3	96	84.9-99.5

Key: *Ax* Assessment, *AUC* area under the curve, *CC* correctly classified, ‘Early’ refers to clinical assessment between 30-32 weeks postmenstrual age, *HNNE* Hammersmith Neonatal Neurological Examination, *NPV* negative predictive value, *PPV* positive predictive value, *Se* sensitivity, *Sp* specificity, ‘TEA’ refers to clinical assessment at term equivalent age, *95% CI* 95% confidence interval.
